# Development of an evaluation indicator system for wound care quality based on the structure-process-outcome model: a mixed-methods study

**DOI:** 10.1186/s12913-026-14823-5

**Published:** 2026-06-03

**Authors:** Yinfeng Hu, Caifei Li, Liqiong Zhou, Yanxia Chen

**Affiliations:** 1 Department of Rheumatology and Immunology, South China Hospital, Medical School, Shenzhen University, Shenzhen, China; 2 Nursing Department, The Third Affiliated Hospital of Sun Yat-Sen University, Guangzhou, China

**Keywords:** Nursing quality indicators, Acute wounds, Chronic wounds, Diabetic foot, Pressure injuries, Lower extremity venous ulcers, Nursing evaluation, Mixed methods

## Abstract

**Background:**

As population aging accelerates and the burden of chronic diseases increases, the incidence of chronic wounds such as diabetic foot ulcers and pressure injuries continues to rise. This trend not only worsens patient outcomes and quality of life but also strains the healthcare system. Traditional wound care assessments rely on isolated indicators, lack systematic monitoring, and fail to provide comprehensive evaluation throughout the care process, thereby limiting improvements in nursing quality. Based on Donabedian’s structure-process-outcome framework, a thorough evaluation system can integrate key elements and multidimensional factors in wound care, supporting evidence-based, standardized nursing management.This study aims to develop a wound care quality evaluation indicator system designed to be clinically applicable and tailored to China’s healthcare context, with the goal of promoting standardized practices and improved service quality.

**Methods:**

This study used the “structure-process-outcome” model to develop a wound care quality evaluation system. In January 2025, initial indicators were identified via literature review and theoretical analysis. In February, semi-structured interviews refined and expanded the indicator pool. A preliminary draft was finalized after an internal team discussion. From March to April 2025, two rounds of expert consultation were conducted, with 27 experts participating in each via email. Data were analyzed using mean ± Standard Deviation (SD), coefficient of variation, and Kendall’s W. Indicator weights were determined using the Analytic Hierarchy Process, yielding a final system with 3 first-level, 9 s-level, and 25 third-level indicators.

**Results:**

The effective response rates for both rounds of the questionnaire survey reached 100%. The authority coefficients for the experts were 0.874 and 0.889, respectively, indicating high expert reliability. Kendall’s coefficient of concordance ranged from 0.169 to 0.181 in the first round and from 0.170 to 0.192 in the second round, reflecting a gradual improvement in consensus among experts. Ultimately, a wound care quality evaluation indicator system was established, comprising 3 first-level indicators, 9 s-level indicators, and 25 third-level indicators.

**Conclusions:**

This indicator system is scientifically rigorous and reliable. It demonstrates theoretical potential for clinical applicability, as it is aligned with wound care practices and nursing management to objectively assess wound management quality. However, its practical operability and real-world applicability require further validation through clinical pilot studies.

**Clinical trial number:**

Not applicable.

**Supplementary Information:**

The online version contains supplementary material available at 10.1186/s12913-026-14823-5.

## Introduction

As the population ages and chronic diseases evolve, the incidence of chronic wounds has risen steadily, with conditions such as diabetic foot ulcers, pressure injuries, and venous leg ulcers becoming a significant global public health challenge [1, 2]. Research indicates that the worldwide incidence of pressure injuries among general hospital inpatients is 12.8% [3]. The prevalence of diabetic foot disease ranges from 2% to 4% in developed countries, but reaches 8.1% in China [4]. Recurrence rates are high, at 40% within 1 year and 65% within 3 years after wound healing [5].

Wound care is a complex clinical process influenced by multiple factors and stages. Its success depends not only on wound severity but also on caregivers’ assessment skills, standardized intervention protocols, and institutional support. This fragmented assessment model cannot accurately reflect the actual value of nursing work or identify quality gaps, limiting improvements in wound care competencies. A scientific, systematic, and practical evaluation framework is therefore essential to enhance clinical standards and support refined management. The quality of wound care directly and clinically significantly influences key patient outcomes, such as time to wound closure, restoration of functional capacity, health-related quality of life, and mortality, as well as the efficient utilization of healthcare resources [6, 7].

In China, however, wound care quality management faces three main challenges. First, the quality management system is underdeveloped; in some institutions, specialized nursing roles receive insufficient attention, resulting in weak administrative support and limited oversight [8]. Second, the lack of standardized assessment indicators results in fragmented data that are difficult to aggregate, hindering systematic evaluation [9]. Third, current clinical evaluation frameworks focus on outcomes rather than processes. At the same time, evidence-based research emphasizes “wound healing,” while key process aspects, such as “pain management,” are often overlooked [10], making it challenging to identify priorities for improvement.

To address the identified bottlenecks, an urgent systematic evaluation framework is needed. The Structure-Process-Outcome (SPO) model, developed by Donabedian, provides a foundational approach to assessing healthcare quality [11]. It defines structure as the basis for quality, process as the core of care delivery, and outcome as the measure of Effectiveness, three interrelated and sequential components that form a coherent, cause-and-effect evaluation system. Applying this model to wound care can overcome limitations of traditional methods, enabling comprehensive assessment across the entire care continuum, from resource allocation to patient outcomes. The Donabedian model has been widely used in nursing and shown to improve care quality [12, 13]. A systematic review shows that nursing quality indicators based on this model improve patient outcomes [12]. Yet, it has not been systematically applied to develop a standardized wound care quality indicator. Meanwhile, the widely used Bates-Jensen Wound Assessment Tool focuses on detailed wound characteristics [13] but does not integrate key elements, such as tissue type, exudate, and wound edge, into a broader quality framework that spans inputs to outcomes. Therefore, combining the SPO model with specialized tools, such as Bates-Jensen, is essential to link clinical wound care with comprehensive quality management.

This study addresses a critical gap in China’s wound care quality management: the absence of a rigorously developed, evidence-informed quality indicator system grounded in the internationally recognized SPO framework, also known as the Donabedian model. The central research question is: Which clinically relevant, scientifically valid, and measurable indicators should constitute a comprehensive wound care quality indicator system for the Chinese healthcare context? Guided by frontline clinical practice and methodological rigor, the resulting system is designed to enable systematic assessment, benchmarking, and continuous quality improvement across the full continuum of wound care, including assessment, prevention, treatment, and outcome evaluation. Primarily intended for use in acute-care hospitals and specialized chronic wound clinics, the system supports internal quality monitoring, peer review, and external accreditation. Its international relevance stems from its dual grounding: adherence to the globally accepted SPO theoretical framework and strict compliance with the Delphi methodology, ensuring both contextual fidelity to China’s practice realities and methodological transparency for global adaptation and validation.

## Methods

The indicator system developed in this study is grounded in two complementary evidence sources: a systematic literature review and semi-structured interviews with clinical healthcare professionals. These evidence sources were systematically integrated and applied during phase 1 of the research. In phase 2, expert consensus on the indicator system was established through a structured Delphi process. This method is widely used in the formulation of quality indicators and consensus guidelines in the medical and healthcare domains [14]. Finally, the Analytic Hierarchy Process (AHP) will be employed to assign weights to each dimension and indicator, thereby establishing a wound care quality evaluation indicator system with a clear structure, quantified weights, and alignment with actual clinical circumstances in China.

### Phase one: construction of the indicator pool

#### The establishment of the research group

A research group was formed comprising one head nurse, one wound care specialist, two international stoma therapists, and two postgraduate students trained in evidence-based methods. The group conducted literature reviews, developed the indicator item pool, drafted the research protocol and initial indicator system, organized interviews, prepared and administered two rounds of expert consultations, refined indicators based on feedback, and performed statistical analysis. To ensure research objectivity, no member of the research group served as a respondent or expert consultant in data collection.

#### Indicator construction based on literature analysis

**Fig. 1:**
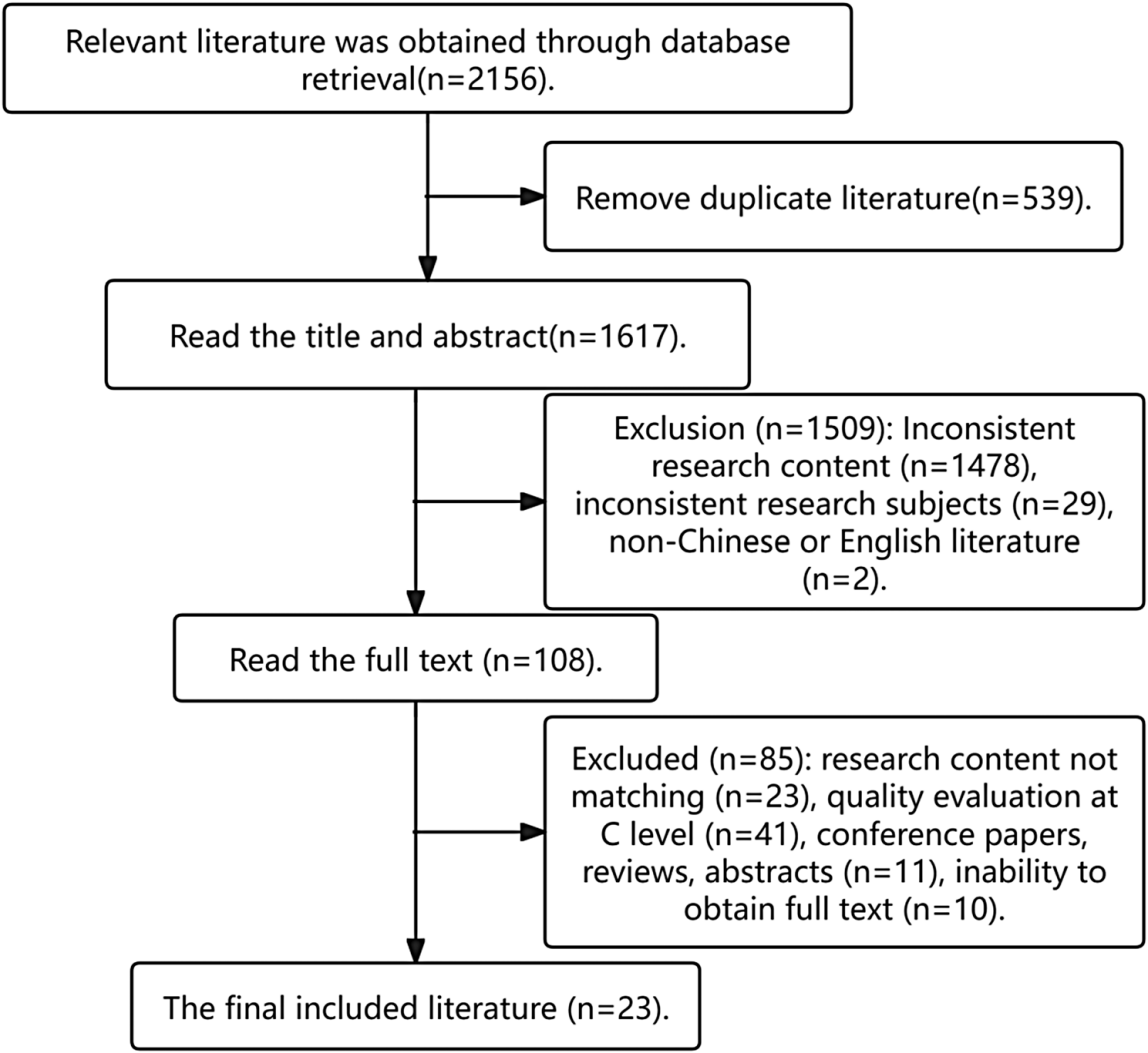
The literature screening flowchart

This study adopted a double-anonymized independent screening strategy. Two researchers, who underwent standardized training, independently conducted the systematic literature search and initial screening. The keywords were including “wound care,” “wound management,” “chronic wounds,” “acute wounds,” “diabetic foot,” “pressure injuries,” “venous ulcers of the lower extremities,” “nursing quality,” “quality indicators,” “quality management,” “quality evaluation,” “quality improvement,” “infection,” “healing,” “therapeutic effect,” and “outcome” in both English and Chinese. The search encompassed authoritative domestic and international databases, including CNKI, Wanfang Database, VIP Database, PubMed, Web of Science, CINAHL, Cochrane Library, and Embase, as well as the official websites of relevant academic institutions. Additionally, the snowball method was employed to trace and retrieve references from the included studies. The search was updated through December 2024. To ensure analytical rigor and reproducibility of results, we prospectively established standardized inclusion and exclusion criteria for literature selection and developed a structured data extraction form. The inclusion criteria were as follows: (1) studies focusing on the development or construction of quality evaluation indicator systems for wound care; (2) research addressing quality improvement strategies in wound management; (3) original research articles published in either Chinese or English that evaluated nursing outcomes and related intervention measures. The exclusion criteria included: (1) non-research materials such as news reports, review articles, and conference abstracts; (2) literature rated at evidence level C or below according to the Johns Hopkins Nursing Evidence-Based Practice framework; (3) duplicate publications or studies for which full-text access was unavailable. Two graduate students independently employed the Johns Hopkins Nursing Practice Evidence Grading System to evaluate the methodological quality and categorize the evidence levels of the included literature [15]. For studies exhibiting discrepancies during the initial screening phase, a third senior expert, uninvolved in the initial screening, conducted an independent review and resolved the disagreements. All discrepant items were systematically discussed in scheduled research group meetings until consensus was achieved. This three-tiered quality control procedure strengthened both the reliability and validity of the literature screening process. A total of 2,156 documents were retrieved. After removing duplicates and conducting multi-level screening, 23 were included in the analysis. For detailed information, please refer to the literature screening flowchart shown in Fig. 1. Based on a synthesis of the literature and guided by clinical relevance, feasibility, and importance, the team developed a preliminary item pool for the Wound Care Quality Evaluation Indicator System.

#### Supplementary indicators derived from clinical healthcare professionals’ interview

In February 2025, we invited 8 clinical healthcare professionals who were not part of the research group, including 2 head nurses and 6 wound care nurses, as respondents to provide qualitative data before the formal Delphi consultation. Inclusion criteria were: (1) over five years in wound and stoma nursing management or over three years of clinical experience; (2) a bachelor’s degree or higher; and (3) a solid understanding of nursing quality indicators. The goal was to identify core elements, develop an initial item pool, and refine the draft questionnaire through cognitive interviews. All respondents provided consent and were recorded, and their responses were transcribed verbatim. The interview outline was initially drafted based on the literature review and was subsequently revised and approved by the four members of the research team. The research group reviewed domestic and international studies on wound care quality assessment, the SPO model, and established nursing quality indicators [8, 12, 16]. Based on this, the interview covered the following questions: (1) What were the main challenges in wound care quality management? (2) What factors most affected wound care outcomes? (3) How could wound care quality be scientifically assessed, and were the current evaluation indicators comprehensive enough? (4) Which nursing interventions were most critical for high-quality care and improved patient outcomes? (5) What core principles should guide nursing practice for wounds of different causes and healing stages? (6) How could multi-professional collaboration in wound care be strengthened? (7) Did you have recommendations for improving the wound care quality evaluation system? The framework analysis method is widely employed in qualitative interview data analysis owing to its structured approach and transparent analytical process [17]. All interviews were conducted by a single researcher who had undergone systematic training in qualitative research methods, ensuring methodological consistency and uniformity in interview style. Audio recordings were transcribed within 24 h of each interview; transcription was performed independently by 2 trained researchers, followed by cross-verification. Each transcript was meticulously aligned with the original audio to guarantee accuracy and completeness. Thematic coding was conducted independently by 2 researchers trained in qualitative coding protocols, using a pre-established analytical framework. Coding discrepancies were first resolved through structured consensus discussions; persistent disagreements were adjudicated by a third senior researcher with over 10 years of experience in qualitative inquiry, thereby strengthening the analysis’s credibility and auditability. The team combined literature-based indicators with semi-structured interview data. For indicators found only in the literature or in interviews, they assessed each against three criteria: theoretical soundness, clinical feasibility, and alignment with treatment guidelines, retaining only those with apparent conceptual necessity and practical justification. This approach ensures rigor and clinical relevance, producing an indicator pool that is both evidence-based and actionable. A literature review yielded 29 initial indicator expressions spanning 11 thematic categories; clinical health professional interviews subsequently refined these into 15 consolidated indicator expressions across 10 themes. After the research team merged, de-duplicated, and standardized, an initial framework for evaluating wound care quality was developed, comprising 3 first-level, 9 s-level, and 26 third-level indicators.

### Phase two: Delphi expert consultation

**Fig. 2:**
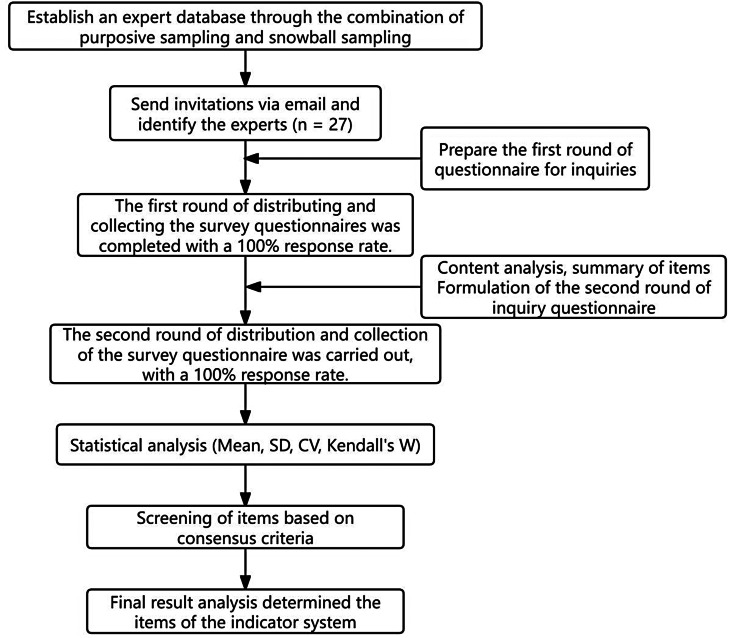
Delphi expert consultation research flowchart

This stage aims to refine the initial item list through a structured Delphi process involving iterative expert consultation, item screening, targeted revision, and consensus attainment. Delphi expert consultation research flowchart (see Fig. 2).

#### Selecting and consulting experts

The number of Delphi experts to be consulted should be limited to 15–30 to prevent homogeneity among the research participants [18]. The clinical health professionals who participated in the semi-structured interviews did not take part in the Delphi expert consultation. In this study, a strategy combining purposive sampling and restricted snowball sampling was adopted to form the expert consultation group. Structured criteria such as institutional level, departmental field, and position hierarchy were used to guide the experts’ recommendations. Combined with cross-source cross-validation and the principle of information saturation termination, the risk of network homogeneity was effectively controlled, thereby constructing an expert group with diverse backgrounds to ensure the comprehensiveness and representativeness of the consensus. The inclusion criteria for expert participants were as follows: (1) more than five years of clinical or managerial experience in wound care or nursing management; (2) possession of a bachelor’s degree or higher, along with a mid-level or higher professional title; (3) voluntary participation in the study and a commitment to actively engage in all stages of the consultation process.27 experts took part in two rounds of Delphi expert consultation via correspondence.

#### Compilation of the expert inquiry questionnaire

Based on evidence-based analysis and expert interviews, a wound care quality evaluation indicator system was established, comprising 3 primary indicators, 9 secondary indicators, and 26 tertiary indicators. An expert inquiry questionnaire was subsequently developed based on this framework. The questionnaire was structured into 3 sections: the first section was the preface, which outlines the research background, objectives, significance, and instructions for completion; the second section contains the expert evaluation form, employing a 5-point Likert Scale to assess the importance of each indicator, ranging from “very unimportant” (1 point) to “very important” (5 points); the third section collects basic information about the experts, aiming to document their professional backgrounds systematically. This included three components: (1) demographic and professional characteristics, such as name, gender, age, highest academic degree, professional title, primary field of practice, and total years of clinical or research experience; (2) the expert’s familiarity with the questionnaire content concerning Coefficient of Self-familiarity (Cs); categorized into five levels—“very familiar,” “familiar,” “somewhat familiar,” “not familiar,” and “not very unfamiliar”—with corresponding quantitative scores of 1.0, 0.8, 0.6, 0.4, and 0.2, respectively; and (3) the basis for the expert’s judgment, denoted by Coefficient of Analysis basis (Ca), which includes four sources: theoretical analysis, practical experience, reference to domestic and international literature, and intuitive judgment. After consulting experts, it was necessary to ask the questionnaire respondents for the specific evidence on which experts based their judgments for the research topic. The result of this inquiry is the expert judgment coefficient (Cₐ), which is calculated as the sum of the assigned values for each judgment basis (Cₐ = Σ v_i_). The specific scoring criteria for each basis are detailed in Table 1. During the expert consultation process, a structured questionnaire will be administered to systematically elicit information on the specific types and provenance of evidence underpinning experts’ judgments. This data will serve as the empirical basis for calculating the expert judgment coefficient. Throughout the study, after analyzing the results of the first-round expert consultation and incorporating feedback, the questionnaire’s content and structure were refined and optimized for the second round of inquiry.

**Table 1:** The assignment of weights for expert judgment

Reference for judging	The degree of impact on the importance of each item		
	Important	General	Unimportant
Practical experience	0.5	0.4	0.3
Theoretical analysis	0.3	0.2	0.1
Domestic and foreign references	0.1	0.1	0.1
Subjective experience	0.1	0.1	0.1

#### The implementation of expert consultation

In April 2025, an expert consultation was conducted through a two-round Delphi-style inquiry. This was a widely utilized circular design for achieving a stable consensus [14]. Questionnaires were distributed via email, and after each round, they were revised based on expert feedback. The experts were subsequently informed of the revisions. The process continued until expert consensus was reached and results stabilized, at which point the inquiry was concluded. The consensus-building process had two stages: statistical analysis and group discussion. In the statistical phase, indicators were assessed using two criteria: an average importance score of 3.5 or higher and a coefficient of variation of 0.25 or lower [19]. Indicators that fail these criteria proceed to group discussion, where evaluation focuses on (1) the score distribution and (2) experts’ written justifications for supporting or opposing the indicator. If the opposition is strong and well-justified, the indicator is removed. If disagreement stems from unclear wording, the indicator is revised to clarify it.

#### Statistical method

Statistical analysis was performed using software, including Microsoft Excel 2021, SPSS 22.0, and Yaahp 12.0. Count data were expressed as percentages, whereas measurement data were summarized using the mean ± standard deviation. The questionnaire response rate was employed to reflect expert engagement; the arithmetic means of familiarity and judgment basis were used to assess expert authority. This study evaluates authority expertise using three parameters: the authority coefficient (Cr), Cs, and Ca, where Cr = (Cs + Ca) / 2 [20]. A Cr value greater than 0.70 indicated acceptable authority, while a value exceeding 0.80 suggested a high level of expert authority [21]. The mean score and standard deviation indicate the concentration of expert opinions. When the mean importance rating for the indicators reaches 3.50, and the coefficient of variation (CV) is less than 0.25, this suggests a high level of consensus among experts [19]. The degree of coordination among experts’ opinions is measured by Kendall’s coefficient of concordance, which ranges from 0 to 1 (*p* < 0.05). A higher value indicates greater consistency in the experts’ judgments [22]. The weights of the indicators were determined using the Analytic Hierarchy Process, with the Saaty scale derived from expert evaluations during the second round of consultations to assess the relative importance of the indicators [23].

**Fig. 3:**
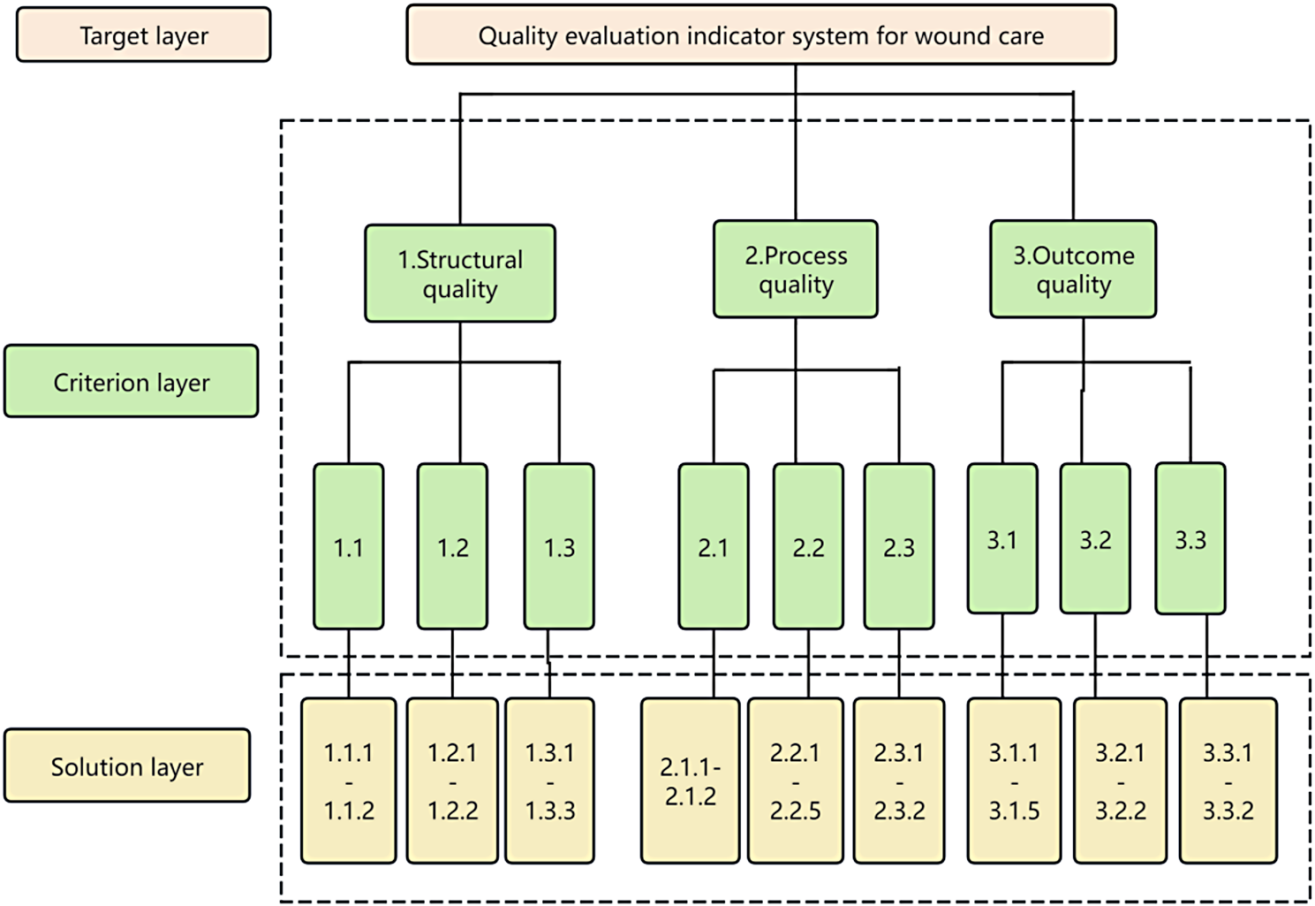
Hierarchical structure model diagram

The hierarchical structure model is presented as follows. As depicted in Fig. 3, in this research, the “wound care quality evaluation indicator system” is designated as the overall evaluation target layer (the highest level), the first - level and second - level indicators as the sub - target layers (criterion layers), and the third - level indicators as the evaluation indicator layers) for the construction of the hierarchical structure model.

## Results

### Expert’s basic information

The panel consisted of 27 experts from Shenzhen and Luoyang, with a mean age of 40.48 ± 4.58 years and an average professional experience of 18.41 ± 4.54 years. The educational backgrounds included two individuals holding doctoral degrees, 12 with master’s degrees, and 13 with bachelor’s degrees. In terms of professional titles, 6 held senior-level positions, 13 held associate senior-level positions, and 8 held intermediate-level positions. Further details are presented in Table 2.

**Table 2:** The basic information of the consulting experts (*N* = 27)

Variables	Classification	Number of people	Percentage
Gender	Male	4	14.8
	Female	23	85.2
Age(years)	30–39	11	40.7
	40–49	14	51.9
	50–60	2	7.4
Education level	Bachelor’s degree	13	48.1
	Master’s degree	12	44.4
	Doctor’s degree	2	7.40
Professional title	Intermediate title	8	29.6
	Associate senior	13	48.1
	Senior	6	22.2
Work Field	Nursing Management	10	37.0
	Wound Nursing	17	63.0
Working years	9–20	18	66.7
	21–30	9	33.3

### The positive coefficients and authority coefficients of experts

In both rounds of the questionnaire survey, 27 questionnaires were distributed, and all were returned, resulting in a 100% response rate. The authority coefficients were 0.874 and 0.889, respectively. Details are shown in Table 3.

**Table 3:** Two rounds of expert authority coefficients

Round	Judgment coefficient	Familiarity coefficient	Authority coefficient
First round	0.896	0.852	0.874
Second round	0.859	0.919	0.889

### Degree of coordination among expert opinions

Kendall’s coefficient of concordance (W) measures the degree of agreement among experts in their evaluations of all indicators. The results show that expert opinions were relatively consistent in both rounds of consultation, with a higher level of coordination observed in the second round, as presented in Table 4 − 1 and Table 4 − 2.

**Table 4−1:** The Kendall’s coefficient of concordance, chi-square value, and degrees of freedom of the first expert consultation

Stage	Indicator	Number of Indicators	Kendall, s W	X2	df	*p*
Round 1	Primary Indicator	3	0.169	9.1	2	0.011
	Secondary Indicator	9	0.181	39.179	8	0.000
	Third-level indicators	26	0.174	122.183	26	0.000

**Table 4−2:** The Kendall’s coefficient of concordance, chi-square value, and degrees of freedom of the second expert consultation

Stage	Indicator	Number of Indicators	Kendall, s W	X2	df	*p*
Round2	Primary Indicator	3	0.182	9.810	2	0.007
	Secondary Indicator	9	0.192	41.465	8	0.000
	Third-level indicators	25	0.170	109.902	24	0.000

### Degree of consensus among expert opinions

A smaller standard deviation corresponds to higher mean values, and a smaller coefficient of variation indicates more concentrated expert opinions. The mean, standard deviation, and coefficient of variation of the first-round expert consultation are presented in Table 5.

**Table 5:** The mean, standard deviation, and coefficient of variation of the first-round expert consultation

Indicator	Importance assignment ()	Coefficient of variation
**1. Structural quality**	**4.30 ± 0.47**	**0.11**
**1.1 Organization and System**	**4.78 ± 0.42**	**0.09**
1.1.1 Formulate written standards for wound assessment and care, operational procedures, and quality evaluation criteria.	4.48 ± 0.51	0.11
1.1.2 Establish a specialized wound-care team or a dedicated position, and precisely define its scope of responsibilities and reporting hierarchy.	4.89 ± 0.32	0.07
**1.2 Personnel Allocation**	**4.37 ± 0.49**	**0.11**
1.2.1The proportion of nurses specializing in wound care, along with their receipt of specialized training and qualification certification status.	4.93 ± 0.27	0.05
1.2.2 The in-service stability and team-building status of core personnel in wound care.	4.59 ± 0.50	0.11
**1.3 Facilities and Equipment**	**4.37 ± 0.49**	**0.11**
1.3.1 The availability rate and integrity rate of wound assessment vehicles, sterile rulers, high-definition cameras, exudate volume measurement tools, and other relevant equipment.	4.78 ± 0.42	0.09
1.3.2 The variety and inventory levels of advanced functional wound dressings adequately address current clinical requirements.	4.37 ± 0.74	0.17
1.3.3 The electronic medical record system is equipped with a structured wound assessment module, which supports image upload and data tracking.	4.74 ± 0.45	0.09
**2. Process quality**	**4.52 ± 0.51**	**0.11**
**2.1 The normativity of the assessment**	**4.48 ± 0.70**	**0.16**
2.1.1 Comprehensiveness of the initial assessment: Upon admission or during consultation, a comprehensive evaluation of the patient’s nutritional status, underlying diseases, and other systemic factors is carried out. Moreover, the wound’s local condition is systematically evaluated according to the Bates-Jensen Wound Assessment and Outcomes Classification Scale.	4.63 ± 0.56	0.12
2.1.2 Dynamic assessment continuity: Develop and implement continuous, individualized assessment plans tailored to the distinct phases of wound healing—specifically, the inflammatory phase, the proliferative phase, and the remodeling phase.	4.63 ± 0.49	0.11
**2.2 Appropriateness of intervention measures**	**3.96 ± 0.44**	**0.11**
2.2.1 The Timing and method of debridement selection: Appropriate debridement should be selected and carried out based on the type of tissue in the wound bed.	4.48 ± 0.51	0.11
2.2.2 The rationale for dressing selection: Dressings with moisture-retentive or absorbent properties should be selected rationally according to the volume of exudate and the stage of the wound.	4.30 ± 0.47	0.11
2.2.3 Effectiveness of infection and inflammation control: Prompt recognition of signs of disease coupled with the timely implementation of effective interventions.	4.52 ± 0.51	0.11
2.2.4 Pain management and patient education: Conduct regular assessments and management of wound-related pain, and offer tailored education to patients and their families.	4.85 ± 0.46	0.09
**2.3 Recording and Communication**	**4.59 ± 0.57**	**0.12**
2.3.1 The key information regarding wounds—including quantity, dimensions, anatomical location, tissue type, and presence of exudate—is documented accurately and objectively.	4.07 ± 0.73	0.18
2.3.2 timeliness of communication: In the event of a deterioration in the wound condition or the emergence of complex problems, communicate promptly with the doctor or the multidisciplinary team.	4.89 ± 0.32	0.07
**3. Outcome quality**	**4.70 ± 0.47**	**0.10**
**3.1 Local outcome of the wound**	**4.30 ± 0.47**	**0.11**
3.1.1 Reduction rate of wound area or volume	4.74 ± 0.59	0.12
3.1.2 The assessment of granulation tissue health is based on its color and extent of coverage.	4.70 ± 0.61	0.13
3.1.3 Epithelialization continues to advance.	4.74 ± 0.53	0.11
3.1.4 Necrotic tissue clearance rate.	4.63 ± 0.69	0.15
3.1.5 Regulate the exudate volume to achieve a balanced state.	4.96 ± 0.19	0.04
3.1.6 The wound edges were assessed for the presence of epithelial creep and the absence of unfavorable conditions, such as maceration and rolled edges.	4.89 ± 0.32	0.07
**3.2 The incidence of complications**	**4.52 ± 0.51**	**0.11**
3.2.1The incidence rate of wound infection.	4.89 ± 0.32	0.07
3.2.2The incidence rate of delayed wound healing or non-healing.	4.59 ± 0.50	0.11
**3.3 Overall patient outcomes**	**4.67 ± 0.48**	**0.10**
3.3.1 Wound-related pain was assessed using the Numerical Rating Scale (NRS).	4.74 ± 0.53	0.11
3.3.2 Improvement in the patients’ ability to engage in activities and their sleep quality.	4.63 ± 0.56	0.12
3.3.3 The awareness rate of patients regarding wound care knowledge and their satisfaction with it.	4.26 ± 0.45	0.11

Note: The bolded text represents the first and second-level indicators; all other items are third-level indicators

The mean, standard deviation, coefficient of variation, and Weighted value of the second-round expert consultation. See Table 6.

**Table 6:** The mean, standard deviation, coefficient of variation, and Weighted value of the second-round expert consultation

Indicator	Importance assignment ()	Coefficient of variation	Weighted value
**1. Structural quality**	**4.37 ± 0.49**	**0.11**	**0.140**
**1.1 Organization and System**	**4.44 ± 0.70**	**0.16**	**0.268**
1.1.1 Formulate written standards for wound assessment and care, operational procedures, and quality evaluation criteria.	4.22 ± 0.42	0.10	0.009
1.1.2 Establish a specialized wound-care team or a dedicated position, and precisely define its scope of responsibilities and reporting hierarchy.	4.56 ± 0.51	0.11	0.028
**1.2 Personnel Allocation**	**4.03 ± 0.19**	**0.05**	**0.117**
1.2.1The proportion of nurses specializing in wound care, along with their receipt of specialized training and qualification certification status.	4.52 ± 0.51	0.11	0.004
1.2.2 The in-service stability and team-building status of core personnel in wound care.	4.85 ± 0.36	0.07	0.012
**1.3 Facilities and Equipment**	**4.70 ± 0.54**	**0.11**	**0.614**
1.3.1 The availability rate and integrity rate of wound assessment vehicles, sterile rulers, high-definition cameras, exudate volume measurement tools, and other relevant equipment.	4.81 ± 0.48	0.10	0.042
1.3.2 The variety and inventory levels of advanced functional wound dressings adequately address current clinical requirements.	4.70 ± 0.61	0.13	0.017
1.3.3 The electronic medical record system is equipped with a structured wound assessment module, which supports image upload and data tracking.	4.74 ± 0.53	0.11	0.027
**2. Process quality**	**4.74 ± 0.45**	**0.09**	**0.333**
**2.1 The normativity of the assessment**	**4.26 ± 0.45**	**0.11**	**0.200**
2.1.1 An initial comprehensive assessment was conducted upon patient admission or consultation, evaluating systemic factors including nutritional status, comorbidities, psychological condition, and social support. A systematic evaluation of local wound characteristics was conducted simultaneously using the Bates-Jensen Wound Assessment Tool (BWAT) to ensure standardized, reliable documentation.	4.63 ± 0.69	0.15	0.044
2.1.2 Dynamic assessment continuity: Develop and implement continuous, individualized assessment plans tailored to the distinct phases of wound healing—specifically, the inflammatory phase, the proliferative phase, and the remodeling phase.	4.41 ± 0.84	0.19	0.022
**2.2 Appropriateness of intervention measures**	**4.33 ± 0.48**	**0.11**	**0.400**
2.2.1 The Timing and method of debridement selection: Appropriate debridement should be selected and carried out based on the type of tissue in the wound bed.	4.44 ± 0.51	0.11	0.020
2.2.2 The rationale for dressing selection: Dressings with moisture-retentive or absorbent properties should be selected rationally according to the volume of exudate and the stage of the wound.	4.37 ± 0.56	0.13	0.014
2.2.3 Effectiveness of infection and inflammation control: Prompt recognition of signs of disease coupled with the timely implementation of effective interventions.	4.74 ± 0.45	0.09	0.045
2.2.4 Pain Management Implementation Rate.	4.74 ± 0.65	0.14	0.045
2.2.5 Patient Education: Offer customized educational services to patients and their families.	4.15 ± 0.66	0.16	0.009
**2.3 Recording and Communication**	**4.33 ± 0.48**	**0.11**	**0.400**
2.3.1 The key information regarding wounds—including quantity, dimensions, anatomical location, tissue type, and presence of exudate—is documented accurately and objectively.	4.07 ± 0.73	0.18	0.022
2.3.2 timeliness of communication: In the event of a deterioration in the wound condition or the emergence of complex problems, communicate promptly with the doctor or the multidisciplinary team.	4.89 ± 0.32	0.07	0.111
**3. Outcome quality**	**4.78 ± 0.42**	**0.09**	**0.528**
**3.1 Local outcome of the wound**	**4.52 ± 0.51**	**0.11**	**0.297**
3.1.1 Reduction rate of wound area or volume	4.48 ± 0.51	0.11	0.017
3.1.2 The assessment of granulation tissue health is based on its color and extent of coverage.	4.89 ± 0.32	0.07	0.062
3.1.3 Epithelialization continues to advance.	4.63 ± 0.49	0.11	0.026
3.1.4 Necrotic tissue clearance rate.	4.78 ± 0.42	0.09	0.041
3.1.5 Wound-related pain control effect.	4.30 ± 0.72	0.17	0.011
**3.2 The incidence of complications**	**4.30 ± 0.61**	**0.14**	**0.163**
3.2.1The incidence rate of wound infection.	4.78 ± 0.42	0.09	0.058
3.2.2The incidence rate of delayed wound healing or non-healing.	4.70 ± 0.47	0.10	0.029
**3.3 Overall patient outcomes**	**4.74 ± 0.45**	**0.09**	**0.540**
3.3.1 Patient awareness of wound care knowledge.	4.63 ± 0.49	0.11	0.190
3.3.2The satisfaction rate among patients receiving wound care.	4.44 ± 0.51	0.11	0.095

Note: The bolded text represents the first and second-level indicators; all other items are third-level indicators. All consistency ratios (CR) were below 0.1, confirming acceptable consistency

### Indicating indicator refinement or modification across Delphi rounds

#### The initial round of the Delphi process

In the first expert consultation, based on feedback, revisions were made: (1) “patient psychological assessment and social support” was added to third-level indicator 2.1.1; (2) “pain management and patient education” 2.2.4 was split into two indicators—2.2.4 “Pain Management Implementation Rate” and 2.2.5 “patient education”; (3) Due to content overlap between third-level indicator 3.1.3 " Epithelialization continues to advance.” and 3.1.6 " The wound edges were assessed for the presence of epithelial creep and the absence of unfavorable conditions such as maceration and rolled edges.” and because 3.1.5 " Regulate the exudate volume to achieve a balanced state.” reflects an intervention goal rather than an outcome indicator, indicators 3.1.3 and 3.1.5 are removed. (4) " Reduction rate of wound area or volume” was added under third-level indicator 3.1.1. See Table 5 for details.

#### The second round of the Delphi method

In this consultation round, based on expert feedback, the indicator system was revised as follows: (1) Tertiary indicator 3.3.1 “Using the NRS to assess wound-related pain” was merged into secondary indicator 3.1 “Local wound outcomes”; (2) Tertiary indicator 3.3.2 “Improvement in patient activity ability and sleep quality” was removed; (3) Tertiary indicator 3.3.3 was split into two separate indicators: “Knowledge awareness rate” and “Patient satisfaction.” See Table 6 for details.

The research team developed a comprehensive wound care quality evaluation system comprising 3 first-level, 9 s-level, and 25 third-level indicators. This system was created by screening, reorganizing, removing redundancies, combining similar items, and optimizing indicators. For detailed content, please refer to Annex 1. The analytic hierarchy process was used to assign weights through pairwise comparisons of indicator importance.

## Discussion

This study developed a quality evaluation indicator system for wound care through a literature review and semi-structured interviews. Two rounds of Delphi expert consultation, combined with the AHP, were employed to refine and weight the indicators, yielding a comprehensive, systematic, and quantifiable assessment tool. The system demonstrates strong content validity and high expert consensus, offering both a theoretical basis and a practical instrument for evaluating and improving wound care quality. A total of 27 experts from Shenzhen and Luoyang, experienced in wound care, nursing management, or related clinical fields, participated in the event. 70% held senior titles, and over half had more than 15 years of experience in their field. Questionnaire response rate was 100% in both rounds, with 15 experts providing specific suggestions, reflecting strong engagement. Kendall’s W ranged from 0.169 to 0.192, all *p* < 0.05, indicating low but statistically significant agreement among the 27 experts. The significant p-values confirm that the rankings are non-random; the low W values reflect genuine heterogeneity—expected given the panel’s multidisciplinary composition, such as practice settings and experience levels. Rather than a methodological limitation, this low agreement empirically confirms the persistence of clinical equipoise across expert groups. In the Delphi process, such divergence is valuable: it highlights items needing more evidence or iterative refinement. Pre-specified criteria determined the final consensus; for example, the coefficient of variation (CV) was less than 0.25 rather than a high Kendall’s W, ensuring that disagreements were transparently reported rather than statistically masked.

The wound care quality evaluation indicators are comprehensive and professionally designed in the present research. The construction process itself is a significant discovery. Expert consensus initially emphasizes the structural foundation (weight 0.140). Given China’s uneven distribution of medical resources, forming specialized teams and structured electronic medical record systems is seen as the key starting point for quality improvement. This assessment system maintains structural quality as its core objective while enabling differentiated implementation pathways for resource-constrained institutions. Specifically, professional capacity building can be advanced through the designation of core liaison nurses, supported by remote specialist alliances and regularly scheduled training programs to facilitate knowledge sharing and skill transfer. Concurrently, the information infrastructure may be incrementally strengthened, beginning with standardized electronic documentation, and prioritized to ensure the structured capture and auditability of critical clinical data. Guided by the principle of “unified objectives, flexible implementation,” this design empowers diverse institutions to initiate sustainable nursing quality improvement within their existing operational and resource constraints, thereby preventing delays in quality advancement attributable to limited capacity. In the process dimension (weight 0.333), standardized assessment and evidence-based intervention emerge as core elements. The study successfully converts components of the Bates-Jensen tool into measurable clinical indicators, such as adherence to debridement guidelines. Notably, pain management and patient education have advanced from peripheral to core indicators, reflecting a shift in nursing philosophy from “wound-centered” to “patient-centered.” Outcome quality carries the highest weight (0.528), consistent with global and domestic research [24–27]. It includes key indicators such as healing and complication rates, as well as patient satisfaction, emphasizing a patient-centered approach. The framework also aligns with China’s National Nursing Development Plan 2021–2025 [27], particularly its goals of advancing high-quality services and innovative models. By integrating international standards with national policy, the system demonstrates strong coherence in design, validity, and responsiveness.

This system is designed to align closely with clinical practice, with the aim of offering high operability and practical guidance. First, the indicators are derived from clinical needs: structural indicators help hospital administrators optimize resource allocation; process indicators, based on the Bates-Jensen tool [28], provide nurses with a standardized framework for wound assessment, debridement, infection control, and dressing selection—reducing variability and improving care consistency. Second, as a dynamic, quantifiable tool, the system enables nursing managers to identify service gaps by monitoring process compliance and outcome trends. For example, delayed healing due to inadequate debridement or poor patient adherence from insufficient education can be quickly detected and corrected. Finally, the system establishes a closed-loop mechanism that translates national initiatives, such as “high-quality nursing” and “continuous quality improvement,“ [29] into actionable practices and measurable outcomes, supporting the shift from experience-based to standardized, evidence-informed wound care and demonstrating its clinical relevance and value.

The indicator system developed in this study provides a ready-to-use framework for clinical quality monitoring and improvement. It can be transformed into a structured audit checklist to routinely assess resource allocation (structure), nursing procedure standardization (process), and patient outcomes (results). Key indicators can also be integrated into hospital performance dashboards for real-time, visual quality monitoring and feedback. To verify the validity and practicality of the indicators, future studies should utilize real-world data. For instance, multicenter electronic health record (EHR) data can be analyzed to examine the relationship between process and outcome indicators. The long-term integration of core indicator components, such as standardized assessment items, into structured EHR modules is essential for automated data collection, calculation, and continuous monitoring. This integration is crucial for transitioning the system from theoretical evaluation to routine clinical practice.

This study is based on Donabedian’s “structure-process-outcome” model and has constructed a systematic evaluation index system for wound care quality. The indicators were selected through a multidisciplinary Delphi method to ensure rigor; however, it should be noted that the Delphi method has consensus pressure, which may weaken the forward-looking perspective. The consensus reached is actually a compromise result of the group and should be cautious when interpreting this methodological limitation. The theoretical framework and core dimensions of this index system have international universality and can serve as the basis for cross-national comparisons; however, the specific indicators, operational definitions, data sources, and evaluation thresholds are highly dependent on local medical resources, information systems, and clinical culture. Therefore, when applying it across regions, the strategy of “framework transplantation and content adaptation” should be adhered to - retaining the three-dimensional structure, adapting the indicators through local expert consensus, and balancing scientificity and feasibility. This study not only provides a practical evaluation tool but also lays the foundation for subsequent cross-context verification and promotion.

## Limitation

This study has the following limitations. Firstly, this study employed the Delphi method. Although rigorous expert selection, clear background information, and the establishment of preset consensus standards enhanced methodological rigor, the reliability of the results depends heavily on the composition, professional backgrounds, and representativeness of the expert panel. Anonymous multi-round feedback may trigger “consensus pressure”, causing experts to conform to the median due to group pressure, authority compliance, or avoidance of controversy, resulting in a “centripetal effect”. Minority but reasonable viewpoints may be diluted or abandoned, leading to “false consensus” or “conformity bias” and weakening the comprehensiveness and innovation of the indicator set, and missing indicators with clinical value that do not reach a high consensus level. Therefore, the final indicator set reflects the compromise result reached through expert interaction under a specific method. Future research can be supplemented by combining qualitative, non-anonymous discussions to verify this, thereby addressing the issue of viewpoint simplification caused by the purely anonymous process. Secondly, the expert panel’s geographic and institutional diversity was limited. We used purposive and snowball sampling, yielding highly qualified experts, but mostly from Luoyang and Shenzhen. While these cities reflect typical northern and southern urban healthcare settings, they underrepresent western China and rural areas, where key factors, such as the availability of wound care nurses, access to wound care products, and patient travel distances, differ significantly. As a result, the indicator system may reflect regional practice patterns and requires further empirical validation for national applicability, especially in resource-constrained settings.Thirdly, this study did not conduct a group analysis of expert scores by demographic characteristics. Although overall consistency was high, future studies with a larger sample size can examine the applicability differences of the indicator system across institutional levels, regions, or professional fields, providing more precise guidance for local implementation. Given that the expert group includes both nursing managers and clinical nurses, future research can examine whether there are differences in the perceived importance of these indicators between the two groups. At the grassroots level, striving for comprehensiveness might lead to increased complexity and insufficient attention to long-term results, such as patient functional status.Future research should expand the “outcome” dimension to include long-term functional recovery, specifically the return to activities of daily living (ADLs) and to work or usual activities. Validated indicators for these outcomes would move beyond immediate satisfaction and wound closure, offering a more comprehensive measure of wound care quality. In future practical applications, there may also be issues such as high data-collection costs and difficulties in quantifying specific indicators. It is recommended that users select core indicators or make appropriate simplifications based on resource conditions in particular scenarios. Fourthly, although the study followed scientific methods, its broader applicability in different medical environments still needs to be further verified. Multicenter, large-sample studies will be conducted to test the sensitivity of these indicators in predicting actual clinical outcomes.

## Conclusion

The wound-care quality evaluation indicator system developed in this study provides a systematic tool for clinical practice and quality management. It should be integrated into routine quality control and electronic medical record systems, with a continuous monitoring mechanism established for process indicators. Staff training should focus on standardized programs centered on the indicator to enhance wound assessment and treatment skills. During implementation, clear definitions of data-collection frequency and operational criteria must be established for structure, process, and outcome indicators to ensure data comparability and timeliness. This system supports the standardization and consistency of wound care services. Future research will focus on four priorities. First, clinical pilot studies in hospitals will assess the system’s operability, measuring assessment time, user acceptance, and data completeness. Second, the indicator system will be validated across diverse geographic settings, progressing from western tertiary hospitals to secondary and county hospitals, then to rural health centers, to test its generalizability across varying resource contexts. Third, based on validation results, either a nationally applicable version or region-specific adaptations, such as a simplified core set for rural areas, will be developed. Fourth, multi-center, large-sample studies will compare this system with existing tools and test the sensitivity of its indicators in predicting clinical outcomes, such as wound-healing rates.

## Electronic Supplementary Material

Below is the link to the electronic supplementary material.

Supplementary Material 1

Supplementary Material 2

Supplementary Material 3

Supplementary Material 4

Supplementary Material 5

Supplementary Material 6

## Data Availability

The data of this study can be provided by the corresponding author upon reasonable request.
