# Supplementary material for: Development of an evaluation indicator system for wound care quality based on the structure-process-outcome model: a mixed-methods study
Source: BMC Health Serv Res. 2026 Jun 3;26:1075. doi: 10.1186/s12913-026-14823-5 (PMC13455128; doi:10.1186/s12913-026-14823-5)
Supplement: Supplementary file 1 [file 12913_2026_14823_MOESM3_ESM.pdf]

Introduction and Process of Analytic Hierarchy Process (AHP)

The Analytic Hierarchy Process (AHP) is a systematic and hierarchical analysis method that combines qualitative and quantitative approaches. It is highly practical and effective in dealing with complex decision-making problems. The basic steps include establishing a hierarchical structure model, constructing pairwise comparison matrices, calculating the weight vector and conducting consistency checks, and computing the combined weight vector. In this paper, AHP is employed to determine the weights of the indicators. The specific process and steps are as follows:

（1）Establish a hierarchical structure model

This paper employs the Analytic Hierarchy Process (AHP) model. In this paper, the AHP is divided into four levels: the goal level, the first-level indicator level, and the second-level indicator level. The specific hierarchical framework is shown in the table.

Table 1 Hierarchical Structure Framework Table

First level indicator	Secondary indicator
A	A1
	A2
	A3
B	B1
	B2
	B3
C	C1
	C2
	C3

Note：A=Structural quality　B=Process quality　C=Outcome quality

A1=Organization and System　A2=Personnel Allocation　A3=Facilities and Equipment B1=The normativity of the assessment　B2=Appropriateness of intervention measures B3=Recording and Communication　C1=Local outcome of the wound　C2=The incidence of complications　C3=The incidence of complications

（2）Establish a judgment comparison matrix

To determine the proportion of a single indicator at a certain level relative to the upper-level indicator (the target level or the first-level indicator level), the average importance score of each indicator given by experts in the second round of the questionnaire survey is

used to assign values to the importance levels of the Satty scale. Indicators at the same level are compared, and pairwise comparison matrices are constructed.The Satty scale values are shown in the table.

Table 2 Satty Relative Importance Scale

Satty scale value	Mean difference in importance ratings	Satty scale value	Mean difference in importance ratings	Significance
1	X-Y=0.0	1		Equal importance
2	0.00<X-Y≦0.25	1/2	-0.25<X-Y≦0.00	
3	0.25<X-Y≦0.50	1/3	-0.50<X-Y≦−0.25	Moderate importance
4	0.50<X-Y≦0.75	1/4	-0.75<X-Y≦−0.50	
5	0.75<X-Y≦1.00	1/5	-1.00<X-Y≦−0.75	Obvious importance
6	1.00<X-Y≦1.25	1/6	-1.25<X-Y≦-1.00	
7	1.25<X-Y≦1.50	1/7	-1.50<X-Y≦-1.25	Strong importance
8	1.50<X-Y≦1.75	1/8	-1.75<X-Y≦-1.50	
9	X-Y＞1.75	1/9	X-Y＜11.75	Extremely Important

Note :X and Y represent the average importance scores of two different indicators at the same level

By making pairwise comparisons of each two elements in the matrix according to the scale in the above table *, a n-order comparison judgment matrix A can be obtained.

n-order judgment matrix

$$A_{n\times n} = \begin{bmatrix} a_{11} & a_{12} & a_{1..} & a_{1n} \\ a_{21} & a_{22} & a_{2..} & a_{2n} \\ a_{..} & a_{..} & a_{..} & a_{..} \\ a_{n1} & a_{n2} & a_{n..} & a_{nn} \end{bmatrix}$$

（3）Calculate the relative weights of the judgment matrix.

The most commonly used methods for calculating the maximum eigenvector of a pairwise comparison matrix are the sum-product method and the root-square method. This paper adopts the sum-product method. The specific calculation steps of the sum-product method are as follows:

①Transform the matrix $A=(a_{ij})_{n\times n}$Each column vector is normalized to obtain $B=(b_{ij})_{n\times n}$

$$b_{ij} = \frac{a_{ij}}{\sum_{i=1}^{n} a_{ij}} \quad (1)$$

②Sum the row vectors of the normalized matrix B.

$$M_i = \sum_{j=1}^{n} b_{ij} \quad (2)$$

③Transform the vector $M=\begin{bmatrix} M1 \\ M2 \\ \cdots \\ M_n \end{bmatrix}$Perform normalization

$$W_i = \frac{M_i}{\sum_{i=1}^{n} M_i} \quad (3)$$

Obtain the eigenvectors $W_i = \begin{bmatrix} W1 \\ W2 \\ \cdots \\ W_n \end{bmatrix}$

④Calculate the maximum eigenvalue λ_{max}

$$\lambda_{max} = \frac{1}{n}\sum_{i=1}^{n} \frac{[AW]_i}{W_i} \quad (4)$$

（4）Consistency check of judgment matrix

①Calculate the consistency index.

$$\mathrm{CI} = \frac{\lambda_{max}-n}{(n-1)} \quad (5)$$

②Calculate the consistency ratio

$$CR = \frac{CI}{RI} = \frac{\lambda_{max}-n}{(n-1)RI} \quad (6)$$

RI is the randomness index. Its specific values are shown in Table *.

Table * Average Random Consistency Index RI Values of Judgment Matrix

Matrix order	1	2	3	4	5	6	7	8	9	10	11	12

RI	0	0	0.52	0.89	1.12	1.26	1.36	1.41	1.46	1.49	1.52	1.54

The standard for consistency check: For judgment matrices with n < 3, they always have complete consistency; for judgment matrices with n ⩾ 3, if the calculated CR < 0.1, it is considered that the matrix meets the requirements and the consistency check is passed; otherwise, the scale values of the elements need to be readjusted and the matrix reconstructed until it meets the requirements.

4.2.1Calculation of the Weights of First-level Indicators

Based on the above-mentioned method for constructing the judgment matrix, the differences in the mean values of the importance of each first-level indicator in this study were substituted to construct the judgment matrix composed of the first-level indicators and the weight calculation results are as follows:

Table 1: Judgment Matrix of First-level Indicators

S	A(Ⅰ)	B (Ⅱ)	C (Ⅲ)
A(Ⅰ)	1	1/3≈ 0.333	1/3≈ 0.333
B(Ⅱ)	3	1	0.5
C(Ⅲ)	3	2	1

According to Formulas 1-6, the weight and consistency test of the judgment matrix are conducted. The calculation process is as follows:

Table Weight and consistency calculation results

Ealuation indicator	Weight Wi	λmax	CI	CR	consistency check
A(Ⅰ)	0.1396				
B(Ⅱ)	0.3325	3.0536	0.02681	0.0516	√
C(Ⅲ)	0.5278				

4.2.2 Calculation of Weights for Secondary Indicators

According to the same calculation steps of the first-level indicator layer, the judgment matrix and weight calculation results of the second-level indicator layer are as follows:

（1）Calculation of indicator weights within Group A

（2）Judgment matrices in Tables A1-A3

A	A1 （Ⅰ-1）	A2（Ⅰ-2）	A3 （Ⅰ-3）
A1（Ⅰ-1）	1	3	1/3≈ 0.333
A2（Ⅰ-2）	1/3≈ 0.333	1	0.25
A3（Ⅰ-3）	3	4	1

The results of weight and consistency calculation are as follows:

Table: Results of Weight and Consistency Calculation

Evaluation indicator	Wight Wi	λmax	CI	CR	consistency check
A1（Ⅰ-1）	0.2684				
A2（Ⅰ-2）	0.1172	3.0735	0.03676	0.0707	√
A3（Ⅰ-3）	0.6144				

（3）Calculation of indicator weights within Group B

Table B1-B2 Judgment Matrix

B	B1	B2	B3
B1（Ⅱ-1）	1	0.5	0.5
B2（Ⅱ-2）	2	1	1
B3（Ⅱ-3）	2	1	1

The results of weight and consistency calculation are as follows:

Table: Results of Weight and Consistency Calculation

Evaluation indicator	Wight Wi	λmax	CI	CR	consistency check
B1（Ⅱ-1）	0.20				
B2（Ⅱ-2）	0.40	3.0000	0.0000	0.0000	√
B3（Ⅱ-3）	0.40				

Calculation of the weights of indicators within Group C

Judgment matrices in Tables C1-C3

C	C1	C2	C3
C1（III-1）	1	2	0.5
C2（III-2）	0.5	1	1/3≈ 0.333
C3（III-3）	2	3	1

Table: Results of Weight and Consistency Calculation

Evaluation indicator	Wight Wi	λ max	CI	CR	Consistency check
C1（III-1）	0.297	3.0092	0.0046	0.0088	√

C2（III-2）	0.1634
C3（III-3）	0.5396

Summary table of the judgment matrix of the indicator layer

Parent node	Compare Elements	Simplified judgment matrix	Wight Wi	λ max	CI	CR	Consistency check
A1（Ⅰ-1）	Ⅰ-1-1，Ⅰ-1-2	[1, 0.333, 3, 1]	[0.25, 0.75]	2.000	0	0	√
A2（Ⅰ-2）	Ⅰ-2-1，Ⅰ-2-2	[1, 0.333, 3, 1]	[0.25, 0.75]	2.000	0	0	√
A3（Ⅰ-3）	Ⅰ-3-1，Ⅰ-3-2，Ⅰ-3-3	[1, 2, 2, 0.5, 1, 0.5, 0.5, 2, 1]	[0.4934, 0.1958, 0.3108]	3.0536	0.0268	0.0516	√
B1（Ⅱ-1）	Ⅱ-1-1，Ⅱ-1-2	[1, 2, 0.5, 1]	[0.6667, 0.3333]	2.000	0	0	√
B2（Ⅱ-2）	5 indicators	See the detailed matrix below	[0.1512, 0.1063, 0.3377, 0.3377, 0.0662]	5.1082	0.0271	0.0242	√
B3（Ⅱ-3）	Ⅱ-3-1，Ⅱ-3-2	[1, 0.2, 5, 1]	[0.1667, 0.8333]	2.000	0	0	√
C1（III-1）	5 indicators	See the detailed matrix below	[0.1079, 0.3938, 0.1674, 0.2598, 0.0712]	5.127	0.0318	0.0283	√
C2（III-2）	III-2-1, III-2-2	[1, 2, 0.5, 1]	[0.6667, 0.3333]	2.000	0	0	√
C3（III-3）	III-3-1, III-3-2	[1, 2, 0.5, 1]	[0.6667, 0.3333]	2.000	0	0	√

The judgment matrix of the five indicators under Ⅱ-2

Relative to Ⅱ-2	Ⅱ-2-1	Ⅱ-2-2	Ⅱ-2-3	Ⅱ-2-4	Ⅱ-2-5	Weight
Ⅱ-2-1	1	2	1/3≈ 0.333	1/3≈ 0.333	3	0.1512
Ⅱ-2-2	0.5	1	1/3≈ 0.333	1/3≈ 0.333	2	0.1063
Ⅱ-2-3	3	3	1	1	4	0.3377
Ⅱ-2-4	3	3	1	1	4	0.3377
Ⅱ-2-5	1/3≈ 0.333	0.5	0.25	0.25	1	0.0662

λ max=5.1082, Cl=0.02706, CR=0.0242＜0.1

The judgment matrix of the five indicators under Ⅲ-1

Relative to Ⅲ-1	Ⅲ-1-1	Ⅲ-1-2	Ⅲ-1-3	Ⅲ-1-4	Ⅲ-1-5	Weight
Ⅲ-1-1	1	1/3≈ 0.333	0.5	1/3≈ 0.333	2	0.1079
Ⅲ-1-2	3	1	3	2	4	0.3938
Ⅲ-1-3	2	1/3≈ 0.333	1	0.5	3	0.1674
Ⅲ-1-4	3	0.5	2	1	3	0.2598
Ⅲ-1-5	0.5	0.25	1/3≈ 0.333	1/3≈ 0.333	1	0.0712

λ max=5.1270, Cl=0.03175, CR=0.0283＜0.1

Calculate the final total weight and verify consistency.

The formula for calculating the total weight: Total weight = Criterion weight × Sub-criterion weight × Indicator weightTotal Weight Calculation Table:

Indicator	criteria weight	Sub-criterion weights	indicator weight	Total weight
I-1-1	0.1396	0.2684	0.25	0.1396×0.2684×0.25 =0.00937
I-1-2	0.1396	0.2684	0.75	0.1396×0.2684×0.75 =0.02810
I-2-1	0.1396	0.1172	0.25	0.1396×0.1172×0.25 =0.00409
I-2-2	0.1396	0.1172	0.75	0.1396×0.1172×0.75 =0.01227
I-3-1	0.1396	0.6144	0.4934	0.1396×0.6144×0.4934 =0.04234
I-3-2	0.1396	0.6144	0.1958	0.1396×0.6144×0.1958 =0.01681
I-3-3	0.1396	0.6144	0.3108	0.1396×0.6144×0.3108 =0.02666
II-1-1	0.3325	0.20	0.6667	0.3325×0.20×0.6667 =0.04434
II-1-2	0.3325	0.20	0.3333	0.3325×0.20×0.3333 =0.02217
II-2-1	0.3325	0.40	0.1521	0.3325×0.40×0.1521 =0.02023
II-2-2	0.3325	0.40	0.1063	0.3325×0.40×0.1063 =0.01414
II-2-3	0.3325	0.40	0.3377	0.3325×0.40×0.3377 =0.04491
II-2-4	0.3325	0.40	0.3377	0.3325×0.40×0.3377 =0.04491

Indicator	criteria weight	Sub-criterion weights	indicator weight	Total weight
II-2-5	0.3325	0.40	0.0662	0.3325×0.40×0.0662 =0.00880
II-3-1	0.3325	0.40	0.1667	0.3325×0.40×0.1667 =0.02217
II-3-2	0.3325	0.40	0.8333	0.3325×0.40×0.8333 =0.11084
III-1-1	0.5278	0.2970	0.1079	0.5278×0.2970×0.1079 =0.01692
III-1-2	0.5278	0.2970	0.3938	0.5278×0.2970×0.3938 =0.06174
III-1-3	0.5278	0.2970	0.1674	0.5278×0.2970×0.1674 =0.02625
III-1-4	0.5278	0.2970	0.2598	0.5278×0.2970×0.2598 =0.04073
III-1-5	0.5278	0.2970	0.0712	0.5278×0.2970×0.0712 =0.01116
III-2-1	0.5278	0.1634	0.6667	0.5278×0.1634×0.6667 =0.05750
III-2-2	0.5278	0.1634	0.3333	0.5278×0.1634×0.3333 =0.02874
III-3-1	0.5278	0.5396	0.6667	0.5278×0.5396×0.6667 =0.18984
III-3-2	0.5278	0.5396	0.3333	0.5278×0.5396×0.3333 =0.09492
