# Supplementary material for: Development of an evaluation indicator system for wound care quality based on the structure-process-outcome model: a mixed-methods study
Source: BMC Health Serv Res. 2026 Jun 3;26:1075. doi: 10.1186/s12913-026-14823-5 (PMC13455128; doi:10.1186/s12913-026-14823-5)
Supplement: Supplementary file 2 [file 12913_2026_14823_MOESM4_ESM.pdf]

Development of an Evaluation Indicator System for Wound Care Quality Based on the Structure-Process-Outcome Model： A Mixed-Methods Study

The first round of expert questionnaire by mail

Dear Expert,

Hello! We sincerely invite you to participate in this expert consultation. This research aims to establish a scientific, systematic, and clinically applicable "Wound Care Quality Evaluation Index System" for China. The quality of wound care is directly related to patient safety, rehabilitation progress, and the efficiency of medical resource utilization. The establishment of standardized evaluation tools will help promote the standardization, homogenization, and continuous improvement of wound care services.

This study takes the classic "structure-process-outcome" three-dimensional quality model as its theoretical framework. The structure dimension focuses on the resources and conditions required for wound care, the process dimension focuses on the standardization and appropriateness of nursing practices, and the outcome dimension focuses on changes in patients' health status and the ultimate effectiveness of care. By integrating these three dimensions, it aims to conduct a comprehensive and three-dimensional evaluation of the quality of wound care.

You are a senior expert in wound care, nursing management or related fields, and your professional insights are crucial to this research. This inquiry aims to seek your judgment on the importance of the initially proposed indicators. Instructions for filling out the questionnaire: 1. Questionnaire structure: This questionnaire consists of three parts. The first part is this introduction; the second part is the "Indicator Importance Scoring Table", please score based on your professional experience; the third part is the "Expert Basic Information Survey Form", which is used to understand your professional background.

2. Scoring Method: Please carefully read each indicator in Part Two and mark a " √ " on the corresponding number based on its importance in evaluating the quality of wound care. A 5-point Likert scale is used: 5 = Very important, 4 = Somewhat important, 3 = Moderately important, 2 = Slightly important, 1 = Not important at all.

3. Opinions and Suggestions: After the score column for each indicator item, there is a "Revision Suggestions" column. If you think the expression of this indicator needs to be modified, deleted or merged, or if you have any other valuable suggestions, please write them directly in this column. Any of your opinions are extremely precious to us.

4. Confidentiality Commitment: All information collected in this survey will be used solely for this academic research. We will strictly keep your personal information and responses confidential.

5. Return Method and Time: Please complete and return the questionnaire by [March 15, 2025]. We sincerely thank you for your support!

Part TWO

Expert consultation form: Importance score of indicators (Please mark " √ " in the corresponding column or fill in)

Indicator	The importance of evaluating the quality of wound care					Amending suggestion
	1 =Very unimportant	2 =not very important	3 =Generally important	4 =More important	5 =Most importment	
**1. Structure quality**						
**1.1 Organization and System**						
1.1.1 Formulate written standards for wound assessment and care, operational procedures, and quality evaluation criteria.						

1.1.2 Establish a specialized wound - care team or a dedicated position, and precisely define its scope of responsibilities and reporting hierarchy.						
1.2 Personnel Allocation						
1.2.1The proportion of nurses specializing in wound care, along with their receipt of specialized training and qualification certification status.						
1.2.2 The in - service stability and team - building status of core personnel in wound care.						
1.3 Facilities and Equipment						
1.3.1 The availability rate and integrity rate of wound assessment vehicles, sterile rulers, high - definition cameras, exudate volume measurement tools, and other relevant equipment.						
1.3.2 The variety and inventory levels of advanced functional wound dressings adequately address current clinical requirements.。						
1.3.3 The electronic medical record system is equipped with a structured wound assessment module, which supports image upload and data tracking.						
**2. Process quality**						

**2.1 The normativity of the assessment**						
2.1.1 Comprehensiveness of the initial assessment: Upon admission or during consultation, a comprehensive assessment of the patient's nutritional status, underlying diseases, and other systemic factors is carried out. Moreover, the local condition of the wound is systematically evaluated in accordance with the Bates - Jensen Wound Assessment and Outcomes Classification Scale.						
2.1.2 Dynamic assessment continuity: Develop and implement continuous, individualized assessment plans tailored to the distinct phases of wound healing — specifically, the inflammatory phase, the proliferative phase, and the remodeling phase.						
2.2 Appropriateness of intervention measures						
2.2.1 The timing and method of debridement selection: Appropriate debridement should be selected and carried out based on the type of tissue in the wound bed.						
2.2.2 The rationale for dressing selection: Dressings with moisture-retentive or absorbent						

properties should be selected rationally according to the volume of exudate and the stage of the wound.						
2.2.3 Effectiveness of infection and inflammation control: Prompt recognition of signs of infection coupled with the timely implementation of effective interventions.						
2.2.4 Pain management and patient education: Conduct regular assessments and management of wound-related pain, and offer tailored education to patients and their families.						
2.3 Recording and Communication						
2.3.1 The key information regarding wounds—including quantity, dimensions, anatomical location, tissue type, and presence of exudate—is documented accurately and objectively.						
2.3.2 Timeliness of communication: In the event of a deterioration in the wound condition or the emergence of complex problems, communicate promptly with the doctor or the multidisciplinary team.						
**3. Outcome quality**						
**3.1 Local outcome of the wound**						

3.1.1 Reduction rate of wound area or volume						
3.1.2 The assessment of granulation tissue health is based on its color and extent of coverage.						
3.1.3 Epithelialization continues to advance.						
3.1.4 Necrotic tissue clearance rate.						
3.1.5 Regulate the exudate volume to achieve a balanced state.						
3.1.6The wound edges were assessed for the presence of epithelial creep and the absence of unfavorable conditions such as maceration and rolled edges.						
**3.2 The incidence of complications**						
3.2.1The incidence rate of wound infection.						
3.2.2The incidence rate of delayed wound healing or non - healing.						

**3.3 Overall patient outcomes**						
3.3.1 Wound-related pain was assessed using the Numerical Rating Scale (NRS).						
3.3.2 Improvement in the patients' ability to engage in activities and their sleep quality.						
3.3.3 The awareness rate of patients regarding wound care knowledge and their satisfaction with it.						

Part Three: Expert Information Survey Form

Instructions for filling in the form: To facilitate the analysis of the data, we kindly ask you to assist in filling out the following three forms. Please mark "√" or fill in the corresponding column according to your situation. Only one "√" can be marked for each item based on the degree of influence. We solemnly promise that your personal information will be kept strictly confidential and used only for research purposes.

《Investigation of Basic Information of Experts》

Education background		Degree		Age	

Duty		Pofessional title		Years of working	
Whether the mentor (please ✔)	□ No	□Master's supervisor	□Doctorial tutor		
English level		E-mail			
Work Experience (Please ✔)	Oversea year	□Domestic year		□Other	
Research fields (please tick ✔)	□Wound care	□Nursing education	□Clinical nursing	□Nursing management	□Other
Workplace and Correspondence Address					
Phone number					

《Survey on Experts' Familiarity with Consultation Content》

Degree of familiarity	Most familiar	Familiar	General	Unfamiliar	Most familiar
Self-assessment by experts					

《Expert form-filling judgment basis investigation》

Expert judgment basis	Judgment basis and influence degree of evaluation indicators		
	Big	Middle	Small
Expert judgment basis			
practical experience			
Refer to domestic and foreign research			
subjective judgment			

—— Thank you again for your guidance and assistance.！ ——
