# Supplementary material for: Development of an evaluation indicator system for wound care quality based on the structure-process-outcome model: a mixed-methods study
Source: BMC Health Serv Res. 2026 Jun 3;26:1075. doi: 10.1186/s12913-026-14823-5 (PMC13455128; doi:10.1186/s12913-026-14823-5)
Supplement: Supplementary file 3 [file 12913_2026_14823_MOESM5_ESM.pdf]

Development of an Evaluation Indicator System for Wound Care Quality Based on the Structure-Process-Outcome Model： A Mixed-Methods Study

The second round of expert questionnaire by mail

Dear Expert:

We sincerely thank you for taking the time out of your busy schedule to support the research of this project. With your assistance and guidance, the first round of expert consultation on the index system has been successfully completed. The research team has integrated the valuable opinions of all experts, and after discussion and literature review, has made adjustments and modifications to some of the index system. Now we have entered the second round of expert consultation. We sincerely invite you to score the importance of the revised index system again and look forward to your valuable opinions.

The second round of the questionnaire survey was based on the first round and the statistical analysis results as well as the feedback from all experts. We have made corresponding modifications to the index system

and marked all the modifications and additions in red. The second round of the questionnaire survey includes 3 first-level indicators, 9 second-level indicators, and 25 third-level indicators. It will take you some precious time to complete this questionnaire survey. We are very grateful for your understanding, support and cooperation! This expert questionnaire survey still consists of three parts. The first part is the basic information survey form for the experts. The second part is the questionnaire survey form for the wound care quality indicator system based on structure-process-result.The third part is the survey form for the experts' familiarity with the research content and the basis for their judgment. Your opinions and suggestions will play a very important role in the scientific nature of this topic. Due to the limited research time, we sincerely ask you to reply within one week after receiving the questionnaire. All the information obtained from this questionnaire survey will be used for the research project, and your data and views will be strictly confidential. If you have any questions about this questionnaire, please contact me. Here, we express our sincere gratitude to you and sincerely look forward to receiving any valuable opinions and suggestions from you on this research!

Instructions for Filling Out the Questionnaire:

1. Structure of the Questionnaire: This questionnaire consists of three parts. The first part is this introduction; the second part is the "Importance Rating Table of Indicators", where you are requested to rate based on your professional experience; the third part is the "Expert Basic Information Survey Form", which is used to understand your professional background.

2. Rating Method: Please carefully read each indicator in the second part and mark a " √ " on the corresponding number according to its importance in evaluating the quality of wound care. A 5-point Likert scale is adopted: 5 = Very Important, 4 = Relatively Important, 3 = Moderately Important, 2 = Relatively Unimportant, 1 = Very Unimportant.

3. Suggestions and Opinions: There is a "Modification Suggestions" column after the rating column for each indicator. If you think the expression of the indicator needs to be modified, deleted, or merged, or if you have any other valuable suggestions, please write them directly in this column. Your opinions are extremely precious to us.

4. Confidentiality Commitment: All information from this survey will only be used for this academic research. We will strictly keep your personal information and responses confidential.

5.Return Method and Time: Please complete and return the questionnaire before [April 15, 2025]. We sincerely thank you for your support!

Indicator	The importance of evaluating the quality of wound care					Amending suggestion
	1 =Very unimportant	2 =not very important	3 =Generally important	4 =More important	5 =Most importment	
**1. Structure quality**						
**1.1 Organization and System**						
1.1.1 Formulate written standards for wound						

assessment and care, operational procedures, and quality evaluation criteria.						
1.1.2 Establish a specialized wound - care team or a dedicated position, and precisely define its scope of responsibilities and reporting hierarchy.						
**1.2 Personnel Allocation**						
1.2.1The proportion of nurses specializing in wound care, along with their receipt of specialized training and qualification certification status.						
1.2.2 The in - service stability and team - building status of core personnel in wound care.						
**1.3 Facilities and Equipment**						
1.3.1 The availability rate and integrity rate of wound assessment vehicles, sterile rulers, high - definition cameras, exudate volume measurement tools, and other relevant equipment.						

1.3.2 The variety and inventory levels of advanced functional wound dressings adequately address current clinical requirements.。						
1.3.3 The electronic medical record system is equipped with a structured wound assessment module, which supports image upload and data tracking.						
**2. Process quality**						
**2.1 The normativity of the assessment**						
2.1.1 An initial comprehensive assessment was conducted upon patient admission or consultation, evaluating systemic factors including nutritional status, comorbidities, psychological condition, and social support. A systematic evaluation of the local wound characteristics was simultaneously performed using the Bates-Jensen Wound Assessment Tool (BWAT) to ensure standardized and reliable documentation.						

2.1.2 Dynamic assessment continuity: Develop and implement continuous, individualized assessment plans tailored to the distinct phases of wound healing—specifically, the inflammatory phase, the proliferative phase, and the remodeling phase.						
**2.2 Appropriateness of intervention measures**						
2.2.1 The timing and method of debridement selection: Appropriate debridement should be selected and carried out based on the type of tissue in the wound bed.						
2.2.2 The rationale for dressing selection: Dressings with moisture-retentive or absorbent properties should be selected rationally according to the volume of exudate and the stage of the wound.						
2.2.3 Effectiveness of infection and inflammation control: Prompt recognition of signs of infection coupled with the timely						

implementation of effective interventions.						
2.2.4 Pain Management: Conduct routine assessments and management of wound-related pain.						
2.2.5 Patient Education: Offer customized educational services to patients and their families.						
**2.3 Recording and Communication**						
2.3.1 The key information regarding wounds—including quantity, dimensions, anatomical location, tissue type, and presence of exudate—is documented accurately and objectively.						
2.3.2 Timeliness of communication: In the event of a deterioration in the wound condition or the emergence of complex problems, communicate promptly with the doctor or the multidisciplinary team.						
**3. Outcome quality**						

**3.1 Local outcome of the wound**						
3.1.1 Reduction rate of wound area or volume						
3.1.2 The assessment of granulation tissue health is based on its color and extent of coverage.						
3.1.3 Epithelialization continues to advance.						
3.1.4 Necrotic tissue clearance rate.						
3.1.5Wound-related pain was assessed using the Numerical Rating Scale (NRS).						
**3.2 The incidence of complications**						
3.2.1The incidence rate of wound infection.						
3.2.2The incidence rate of delayed wound healing or non - healing.						
**3.3 Overall patient outcomes**						
3.3.1The awareness rate of patients about wound care knowledge.						
3.3.2The satisfaction rate among patients						

receiving wound care.						

Expert consultation form: Importance score of indicators (Please mark "√" in the corresponding column or fill in)

Part Three: Expert Information Survey Form

Instructions for filling in the form: To facilitate the analysis of the data, we kindly ask you to assist in filling out the following three forms. Please mark "√" or fill in the corresponding column according to your situation. Only one "√" can be marked for each item based on the degree of influence. We solemnly promise that your personal information will be kept strictly confidential and used only for research purposes.

《Investigation of Basic Information of Experts》

Education background		Degree		Age	
Duty		Pofessional title		Years of working	
Whether the mentor (please ✔)	□ No □Master's supervisor □Doctorial tutor				
English level		E-mail			

Work Experience (Please ✔)	Oversea year	□Domestic year		□Other	
Research fields (please tick ✔)	□Wound care	□Nursing education	□Clinical nursing	□Nursing management	□Other
Workplace and Correspondence Address					
Phone number					

《Survey on Experts' Familiarity with Consultation Content》

Degree of familiarity	Most familiar	Familiar	General	Unfamiliar	Most familiar
Self-assessment by experts					

《Expert form-filling judgment basis investigation》

Expert judgment basis	Judgment basis and influence degree of evaluation indicators		
	Big	Middle	Small
Expert judgment basis			
practical experience			
Refer to domestic and foreign research			
subjective judgment			

—— Thank you again for your guidance and assistance.! ——
