# Supplementary material for: Development of an evaluation indicator system for wound care quality based on the structure-process-outcome model: a mixed-methods study
Source: BMC Health Serv Res. 2026 Jun 3;26:1075. doi: 10.1186/s12913-026-14823-5 (PMC13455128; doi:10.1186/s12913-026-14823-5)
Supplement: Supplementary file 4 [file 12913_2026_14823_MOESM1_ESM.docx]

Evaluation Indicator System for Wound Care Quality

| Primary indicator | Secondary indicator | Third-level indicators |
| --- | --- | --- |
| 1. Structural quality | 1.1 Organization and System | 1.1.1 Develop comprehensive written guidelines for wound assessment and care, standard operating procedures, and evidence-based quality evaluation criteria. |
|  |  | 1.1.2 Establish a specialized wound care team or designate a dedicated role with clearly defined responsibilities and reporting lines. |
|  | 1.2 Personnel Allocation | 1.2.1 The proportion of wound care nurses who have received specialized training and hold formal qualification certification. |
|  |  | 1.2.2 The on-the-job stability of core personnel in wound care and the status of team development. |
|  | 1.3 Facilities and Equipment | 1.3.1 The equipment availability and integrity rates for wound assessment vehicles, sterile rulers, high-definition cameras, and fluid leakage measurement tools were consistently maintained at high levels. |
|  |  | 1.3.2 The diverse types and adequate quantities of novel functional wound dressings currently available are sufficient to meet clinical requirements. |
|  |  | 1.3.3 The electronic medical record system incorporates a structured wound assessment module that facilitates image uploads and enables comprehensive data tracking. |
| 2. Process quality | 2.1 The normativity of the assessment | 2.1.1 Initial assessment comprehensiveness: Upon patient admission or consultation, perform a comprehensive evaluation of systemic factors, including nutritional status, underlying diseases, psychological condition, and social support; simultaneously, conduct a systematic assessment of the wound's local status in accordance with the TIME principle. |
|  |  | 2.1.2 Dynamic assessment of continuity: Develop and implement a continuous, individualized evaluation plan tailored to the distinct stages of wound healing, namely the inflammatory phase, the proliferative phase, and the remodeling phase. |
|  | 2.2 Appropriateness of intervention measures | 2.2.1 Timing and method of debridement: Accurately select and implement debridement according to the specific tissue type present in the wound bed(T). |
|  |  | 2.2.2 Dressing selection should be guided by the wound stage and exudate level, with appropriate use of moisture-retentive or absorbent dressings to support optimal healing conditions(M) |
|  |  | 2.2.3 Effectiveness of infection and inflammation control: Prompt recognition of signs of infection and timely implementation of effective interventions(I). |
|  |  | 2.2.4 Pain Management: Systematically evaluate and address pain associated with wounds regularly. |
|  |  | 2.2.5 Patient Education: Provide personalized education to patients and their families. |
|  | 2.3 Recording and Communication | 2.3.1 The accuracy of record-keeping: The key information, such as the number, size, location, tissue type, and exudate of the wounds, is recorded accurately and objectively. |
|  |  | 2.3.2 Timeliness of communication: Promptly communicate with the physician or multidisciplinary team when wound conditions deteriorate or complex clinical issues emerge. |
| 3. Outcome quality | 3.1 Local outcome of the wound | 3.1.1 Reduction rate of wound area or volume, and wound healing rate. |
|  |  | 3.1.2 The assessment of granulation tissue health is based on its color and extent of coverage. |
|  |  | 3.1.3 Rate of necrotic and dead tissue clearance. |
|  |  | 3.1.4 Evaluate the presence of epithelial creep at the wound margin and ensure the absence of maceration, wound edge rolling, or other adverse tissue conditions(E). |
|  |  | 3.1.5 Use the Numeric Rating Scale to assess wound-related pain. |
|  | 3.2 The incidence of complications | 3.2.1 The incidence of wound infection. |
|  |  | 3.2.2 The incidence of delayed wound healing or non-healing。 |
|  | 3.3 Overall patient outcomes | 3.3.1 The awareness rate of wound care knowledge among patients. |
|  |  | 3.3.2 The satisfaction rate of patients with wound care. |
