# Supplementary material for: Development of an evaluation indicator system for wound care quality based on the structure-process-outcome model: a mixed-methods study
Source: BMC Health Serv Res. 2026 Jun 3;26:1075. doi: 10.1186/s12913-026-14823-5 (PMC13455128; doi:10.1186/s12913-026-14823-5)
Supplement: Supplementary file 5 [file 12913_2026_14823_MOESM2_ESM.docx]

Semi-structured interviews based on the "structure-process-outcome" wound care quality indicator system

一、Methods

1. Interviewee

This study adopted purposive sampling method and selected two head nurses and six wound care specialist nurses from the wound and ostomy clinics, endocrinology departments and geriatrics departments of three tertiary grade A hospitals for semi-structured interviews. The information of the respondents is as follows:

Title distribution: 3 senior nurses, and 3 deputy chief nurses.

Years of experience: The years of experience in wound care-related work range from 3 to 16 years.

Sampling criteria: Sampling will cease when the interview information reaches "saturation", that is, when new interviews no longer provide significant new information.

Data Collection

1.1 Interview Outline: Design the interview outline based on the "structure-process-result" theoretical framework. The interview mainly focuses on the following core questions:

(1)What are the main challenges in wound care quality management? (2) What factors most affect wound care outcomes? (3) How can wound care quality be scientifically assessed, and are current evaluation indicators comprehensive enough? (4) Which nursing interventions are most critical for high-quality care and improved patient outcomes? (5) What core principles should guide nursing practice for wounds

of different causes and healing stages? (6) How can multi-professional collaboration in wound care be strengthened? (7) Do you have recommendations for improving the wound care quality evaluation system?

1.2 Implementation process: The interviews were conducted by trained researchers in quiet meeting rooms or offices, each lasting approximately 30 to 50 minutes. With the informed consent of the respondents, the entire interview was recorded and notes were taken simultaneously to capture non-verbal information.

2. Data analysis

After the interview, transcribe the audio materials verbatim into text within 24 hours and anonymize the text. Data analysis will be conducted using the seven-step phenomenological analysis method of Colaizzi:

(1) Familiarize with the materials: Read all the interview records repeatedly to gain an overall sense.

(2) Extract important statements: Extract meaningful statements from the text related to the research questions.

(3) Construct meaning: Code the extracted important statements and distill their implicit meanings.

(4) Aggregate meaning: Gather the coded meanings to form thematic clusters.

(5) Write detailed descriptions: Provide detailed descriptions of the formed themes.

(6) Refine essential themes: Identify and refine themes that reflect the essence of the phenomenon.

(7) Validate: Return the final analysis results to 2-3 respondents for verification to confirm the authenticity and accuracy of the results.

二、Results

Through in-depth analysis of the interview data, we have distilled the following six core themes around the three dimensions of "structure-process-outcome":

Theme 1: Structural Quality, Professional Human Resources Are the Cornerstone of Quality.

Almost all respondents emphasized that having a professionally certified wound and ostomy therapist is the core of providing high-quality care. They are not only the executors of techniques but also educators and quality promoters within the department. N3: "Our hospital has established a wound care team, but there are only one or two core members. Nurses from other departments are willing but lack systematic training, and often feel overwhelmed." This reflects the structural problem of a shortage and uneven distribution of professional talents.

Theme 2: Structural Quality, Accessibility and Standardization of Materials and Equipment

Respondents generally believe that new dressings and negative pressure wound therapy devices, etc., are key to effective care. However, their "accessibility" and "standardized use" are major challenges. N5: "Many effective dressings are not covered by medical insurance, putting a heavy financial burden on patients. We have to make do with less effective options. Moreover, the reserves of dressings vary among different departments, and there is no unified standard."

Theme 3: Process Quality, Systematized Processes for Evaluation and Decision-Making

High-quality wound care begins with a comprehensive and systematic assessment. Respondents emphasized that this not only involves the local condition of the wound (size, depth, exudate, signs of infection), but also the patient's overall condition (nutrition, blood sugar, blood circulation) and psychosocial factors. N8: "The biggest problem is not the inability to change dressings, but the incomplete assessment in the early stage, which leads to incorrect diagnosis and repeated errors in treatment plans. A standardized assessment tool is needed to guide this."

Theme 4: Process Quality, Communication Barriers in Cross-Departmental Collaboration

Wound care, especially for complex wounds, requires the collaboration of multidisciplinary teams (MDTs). During interviews, "poor communication" and "complex referral processes" were frequently mentioned. N6: "To consult the nutrition department, one has to fill out forms, wait for approval, and then wait for them to schedule a time. Sometimes, I feel it's too much trouble and just give suggestions based on my experience, but this actually poses risks." This indicates that there are breaks in the care process at the departmental interface.

Theme 5: Process Quality, Patient Participation and the Challenges of Personalized Health Education

Healthcare workers unanimously agree that patient compliance is a key factor influencing outcomes. However, providing effective and personalized health education poses significant challenges. N1: "You explain it once to a young patient and they remember it, but for elderly patients, those with cognitive impairments, or those with poor family support, even if you explain it repeatedly, the effect is still very poor. How to ensure they can follow through at home is our biggest headache."

Theme 6: Quality of Outcomes, Multi-dimensional Outcome Evaluation Centered on Patients' Feelings

Apart from the objective indicator of "wound healing", respondents highly emphasized patients' subjective feelings. Pain relief and patients' awareness rate of wound care knowledge were repeatedly mentioned. N4: "It's certainly gratifying to see a bedridden elderly person's pressure ulcer heal, but what gives me a greater sense of achievement is when they can sit up, their pain is reduced, and they have a smile on their face. This is the true 'good outcome'."

The results of this semi-structured interview not only verified many items in the previously constructed indicator system, but more importantly, it revealed the complex practical predicaments and humanistic concerns behind the indicators from the first-hand experiences of clinical front-line workers, providing valuable qualitative basis for the weight setting and practical application of the indicator system.
