# Supplementary material for: Development of an evaluation indicator system for wound care quality based on the structure-process-outcome model: a mixed-methods study
Source: BMC Health Serv Res. 2026 Jun 3;26:1075. doi: 10.1186/s12913-026-14823-5 (PMC13455128; doi:10.1186/s12913-026-14823-5)
Supplement: Supplementary file 6 [file 12913_2026_14823_MOESM6_ESM.docx]

**Annex 1**

Evaluation Indicator System for Wound Care Quality

| Primary indicator | Weighted value | Secondary indicator | Weighted value | Third-level indicators | Weighted value |
| --- | --- | --- | --- | --- | --- |
| 1. Structural quality | 0.140 | 1.1 Organization and System | 0.268 | 1.1.1 Formulate written standards for wound assessment and care, operational procedures, and quality evaluation criteria. | 0.009 |
|  |  |  |  | 1.1.2 Establish a specialized wound-care team or a dedicated position, and precisely define its scope of responsibilities and reporting hierarchy. | 0.028 |
|  |  | 1.2 Personnel Allocation | 0.117 | 1.2.1The proportion of nurses specializing in wound care, along with their receipt of specialized training and qualification certification status. | 0.004 |
|  |  |  |  | 1.2.2 The in-service stability and team-building status of core personnel in wound care. | 0.012 |
|  |  | 1.3 Facilities and Equipment | 0.614 | 1.3.1 The availability rate and integrity rate of wound assessment vehicles, sterile rulers, high-definition cameras, exudate volume measurement tools, and other relevant equipment. | 0.042 |
|  |  |  |  | 1.3.2 The variety and inventory levels of advanced functional wound dressings adequately address current clinical requirements. | 0.017 |
|  |  |  |  | 1.3.3 The electronic medical record system is equipped with a structured wound assessment module, which supports image upload and data tracking. | 0.027 |
| 2. Process quality | 0.333 | 2.1 The normativity of the assessment | 0.200 | 2.1.1 An initial comprehensive assessment was conducted upon patient admission or consultation, evaluating systemic factors including nutritional status, comorbidities, psychological condition, and social support. A systematic evaluation of local wound characteristics was conducted simultaneously using the Bates-Jensen Wound Assessment Tool (BWAT) to ensure standardized, reliable documentation. | 0.044 |
|  |  |  |  | 2.1.2 Dynamic assessment continuity: Develop and implement continuous, individualized assessment plans tailored to the distinct phases of wound healing—specifically, the inflammatory phase, the proliferative phase, and the remodeling phase. | 0.022 |
|  |  | 2.2 Appropriateness of intervention measures | 0.400 | 2.2.1 The Timing and method of debridement selection: Appropriate debridement should be selected and carried out based on the type of tissue in the wound bed. | 0.020 |
|  |  |  |  | 2.2.2 The rationale for dressing selection: Dressings with moisture-retentive or absorbent properties should be selected rationally according to the volume of exudate and the stage of the wound. | 0.014 |
|  |  |  |  | 2.2.3 Effectiveness of infection and inflammation control: Prompt recognition of signs of disease coupled with the timely implementation of effective interventions. | 0.045 |
|  |  |  |  | 2.2.4 Pain Management Implementation Rate. | 0.045 |
|  |  |  |  | 2.2.5 Patient Education: Offer customized educational services to patients and their families. | 0.009 |
|  |  | 2.3 Recording and Communication | 0.400 | 2.3.1 The key information regarding wounds—including quantity, dimensions, anatomical location, tissue type, and presence of exudate—is documented accurately and objectively. | 0.022 |
|  |  |  |  | 2.3.2 timeliness of communication: In the event of a deterioration in the wound condition or the emergence of complex problems, communicate promptly with the doctor or the multidisciplinary team. | 0.111 |
| 3. Outcome quality | 0.528 | 3.1 Local outcome of the wound | 0.297 | 3.1.1 Reduction rate of wound area or volume | 0.017 |
|  |  |  |  | 3.1.2 The assessment of granulation tissue health is based on its color and extent of coverage. | 0.062 |
|  |  |  |  | 3.1.3 Epithelialization continues to advance. | 0.026 |
|  |  |  |  | 3.1.4 Necrotic tissue clearance rate. | 0.041 |
|  |  |  |  | 3.1.5 Wound-related pain control effect. | 0.011 |
|  |  | 3.2 The incidence of complications | 0.163 | 3.2.1The incidence rate of wound infection. | 0.058 |
|  |  |  |  | 3.2.2The incidence rate of delayed wound healing or non-healing. | 0.029 |
|  |  | 3.3 Overall patient outcomes | 0.540 | 3.3.1 Patient awareness of wound care knowledge. | 0.190 |
|  |  |  |  | 3.3.2The satisfaction rate among patients receiving wound care. | 0.095 |
